# Possible Treatment Concepts for the Levodopa-Related Hyperhomocysteinemia

**DOI:** 10.1155/2009/969752

**Published:** 2009-09-09

**Authors:** Thomas Müller

**Affiliations:** Department of Neurology, St. Joseph Hospital Berlin-Weissensee, Gartenstr. 1, 13088 Berlin, Germany

## Abstract

The saga of harmful levodopa (LD) in the treatment of Parkinson's disease (PD) resulted from outcomes of animal—and cell culture studies and the clinical observation of motor complication related to the short half life of LD. Further aspects of LD long term application, the LD associated homocysteine increase and its emerging consequences on progression, and onset of neuropsychiatric symptoms and of vascular disease are only partially considered. Therapeutic approaches for this LD-mediated neurotoxic homocysteine increase are vitamin supplementation or LD application with an inhibitor of catechol-O-methyltransferase (COMT). However, forcing central dopamine metabolism further down the methylation path by central blocking of COMT and MAO-B may reduce oxidative stress and homocysteine levels. But it may also increase N-methylation of tetrahydroisoquinolines to neurotoxic N-methylated tetrahydroisoquinolines. These compounds were observed in cerebrospinal fluid and plasma of long term LD-treated PD patients. Therefore LD application with peripheral COMT inhibition may be safer.

## 1. Introduction

**Figure 1:**
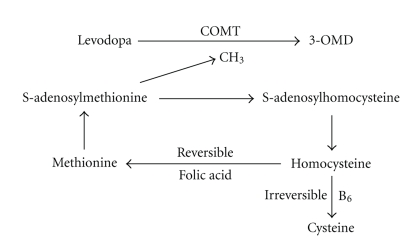
Metabolic pathways of homocysteine generation and degradation in association with levodopa metabolism. COMT=catechol-O-methyltransferase, 3-OMD=3-O-methyldopa, CH_3_=methylgroup.

The discussion of the value of levodopa (LD) for treatment of patients with Parkinson's disease (PD) focused on a putative LD toxicity to date. This saga of harmful LD based on outcomes of animal—and cell culture studies in basic research and the clinical observation of the manifestation of long term motor complications. Therefore the Earlier versus Later L-DOPA (ELLDOPA) trial was designed to answer whether LD is harmful or not. The outcomes showed that LD is anefficacious and well-tolerated compound for treatment of PD patients. This study also provided also some evidence for progression modifying benefits of LD in PD patients, since—after a two wash out period of LD at the end of the trial—the PD patients with prior LD treatment performed significant better than the ones on placebo. One may assume that LD treatment maintained body function and prevented onset of secondary long term changes and adaptation of the body to the manifestation of PD. This may support the concept of an early diagnosis of PD with subsequent early initiation of PD treatment. However a further positive effect of LD may be the growth hormone inducing and thus putative regenerative effects of LD [1]. Growth hormone is under debate as an antiageing compound with multiple efficient regenerative modes of action in the human body [2]. However the ELLDOPA trial lasted 9 months; therefore it could not discuss additional aspects of LD treatment on progression of PD. These points are related to the inhibition of the main LD metabolising enzymes, catechol-O-methyltransferase (COMT), and dopa decarboxylase. Peripheral dopa decarboxylase inhibition (DDI) supports the predominant conversion of LD to its metabolite 3-O-methyldopa (3-OMD) by the ubiquitious enzyme COMT in blood, peripheral tissues and in nigrostriatal neurons (Figure 1). COMT has a broad detoxification potential in humans [3, 4]. In order to prolong the half life and thus to increase brain delivery of LD, COMT-inhibitors were developed as adjuncts to LD/DDI application. Two compounds are currently available, the only peripherally acting entacapone and the additional centrally working tolcapone [5, 6]. COMT inhibition is also under suspicion to prevent motor complications since the rise and fall of LD thus striatal dopamine are less intense during COMT inhibition, that is, entacapone. This supports the concept of continuous dopaminergic stimulation due to a more continuous LD brain delivery [7]. But COMT inhibition has further beneficial effects on the LD-associated homocysteine increase [8]. Long-term LD intake promotes homocysteine elevation, which has an atherosclerosis promoting effect. An occurrence of increased hazard ratios for both ischemic heart and cerebrovascular disease is known in LD/DDI-treated PD patients, who were predominantly treated with LD monotherapy during the LD era [9, 10]. The conversion of LD to 3-OMD via the COMT requires Mg^2+^ as cofactor and S-adenosylmethionine as methyl donor [11]. Thus O-methylation of LD to 3-OMD is associated with conversion of S-adenosylmethionine to S-adenosylhomocysteine and subsequently homocysteine. Elevated homocysteine levels appeared in treated PD patients compared to matched controls [12, 13]. Treated but not previously untreated PD patients showed augmented homocysteine plasma concentrations [14, 15]. The relationship between homocysteine and 3-OMD plasma levels provided further evidence for an impact of LD on homocysteine [16].

## 2. Homocysteine and Arteriosclerosis

Both prospective and case-control studies have shown that an elevated plasma total homocysteine level is an independent risk factor for occlusive vascular disease. Various mechanisms have been suggested for the vascular lesions associated with hyperhomocysteinemia. The redox property of the sulfhydryl group of homocysteine, leading to the formation of reactive oxygen species, is believed to play a pivotal role. This supports substantial impairment of endothelial function and subsequent atherosclerosis [17, 18]. Tie consequence is a vicious circle, since arteriosclerotic disease of striatal cerebral vessels hypothetically results in subsequent onset of increased susceptibility to impaired mitochondrial energy metabolism, oxidative stress, and basal ganglia circuit dysfunction, all of which represent typical, pathophysiologic features of PD [19].

## 3. Cysteine and Arteriosclerosis

Homocysteine is removed either by methylation to methionine or by its irreversible conversion to cysteine (Figure 1). This is a transsulfuration process [20, 21]. The homocysteine derivative, cysteine, is a sulfhydryl-containing amino acid with structural and chemical properties similar to those of homocysteine. Oxidation of cysteine in vitro also promotes several processes considered to be involved in atherogenesis and thrombogenesis. Cysteine has a cytotoxic effect in vitro against several cell types [20]. Cysteine forms an adduct with nitric oxide and may thereby impair endothelial function. Nitric oxide is believed to play a crucial role in the pathogenesis of chronic neurodegenerative processes [20]. High levels of homocysteine cause complex changes in cysteine levels and the overall aminothiol status in plasma [22]. A number of trials showed a close relationship between homocysteine and cysteine metabolism in various disease entities [20]. In plasma, the cysteine concentration is 20-fold higher than the homocysteine level. Rare information is available on cysteine levels in PD patients. Previous trials reported no relevant alterations of cysteine concentrations in PD patients, but moment of blood sampling in relation to intake of LD was not defined in detail in that studies [23–26]. A more recent study defined the moment of blood sampling one hour after LD/DCI intake and divided the cohort of PD patients according to their homocysteine level at the treshold of 15 *μ*mol/L [27]. Only PD patients with an elevation of plasmatic homocysteine above 15 also showed an increase of cysteine plasma levels. This could have been due to the significant higher dosing of daily LD/DCI and the significant higher morning LD/DCI intake of PD patients with a homocysteine concentration above 15 in comparison with the remaining PD patients and the controls. The found significant correlations between morning LD/DCI dosages and cysteine, respectively, homocysteine concentrations, supported this view. Higher LD/DCI intake even seemed to influence cysteinyl-glycine levels due to the significant correlations in patients with elevated homocysteine concentrations [27].

## 4. Cysteinyl-Glycine and Arteriosclerosis

Metabolically interrelated to homocysteine and cysteine is cysteinyl-glycine. In plasma, this thiol species also determines the redox milieu and free radical generation rates. In another neurodegenerative disorder, Alzheimer's disease, cysteinyl-glycine was reduced in plasma. Cysteinyl-glycine is a metabolite of glutathione. Glutathione depletion plays a crucial role in chronic neurodegeneration, since this leads to the accumulation of reactive oxygen species and, ultimately, oxidative stress related, apoptotic cell death, both of which are looked upon as essential pathophysiologic features of PD [22, 28]. All these thiol species—homocysteine, cysteine, and cysteinyl-glycine—exist in plasma in reduced, oxidized, and protein-bound forms, interacting with each other through redox and disulfide exchange reactions. Thus, hyperhomocysteinemia should not be considered as an isolated factor in relation to vascular disease and chronic neurodegeneration. The associated changes in other plasma aminothiols may modulate or even mediate atherogenesis, thrombogenesis, and the neurodegenerative process [20].

## 5. LD-Associated Homocysteine Increase and Clinical Consequences

Homocysteine increase may predispose for neuronal dysfunction and brain atrophy [29, 30]. This toxic impact of homocysteine on neuronal cell death was shown in sural nerves of PD patients and in animal trials [19, 31]. Thus, long-term LD administration may be indirect toxic to neuronal function due to homocysteine elevation [19, 26, 32]. Increase of vascular disease-related thickening of the intima media complex and an elevated risk for onset of neuropsychiatric complications, like depression or deteriorated cognitive function, were also described in association with augmented homocysteine levels in LD/DDI-treated PD patients [33–36]. However the outcomes of more recent trials were only partial confirmatory [37, 38]. This could have been due to a relative low number of participants, design, and duration of these studies. Moreover variations of the distribution of the polymorphism of the Methylenetetrahydrofolate reductase (MTHFR) gene could have differed between the various investigated cohorts. In particular the homozygote MTHFR T/T allele promotes elevated homocysteine levels [33, 39, 40]. Further causes for the controversial outcomes could result from variations of plasma bioavailability of LD. This depends on the gastrointestinal absorption and transport via the gastrointestinal amino acid transporter system. Both of them are influenced by gastric emptying time, which, if reduced, could decrease plasma appearance of LD [41]. Absorption of LD improves with progression of PD and chronic LD intake. As a result, this may support the risk of a LD/DDI-associated homocysteine increase [42, 43]. A further influence on LD metabolism may result from body weight. It is known that low body weight is associated with higher plasma LD levels [44]. All these components, related with LD metabolism, could also have influenced outcomes of trials on the clinical long-term consequences of the LD/DDI-induced homocysteine increase in PD patients. Homocysteine levels were mostly determined one time only in those investigations. A more recent study describes plasma metabolism of LD as essentially contributing component for homocysteine elevation after one time LD/DDI administration [45].

## 6. Homocysteine Increase and LD Degradation

LD metabolism is mainly influenced by the enzyme activities of dopadecarboxylase and COMT; the efficacy of inhibitors of these enzymes might also impact homocysteine levels. In this respect, application of LD only with a DDI supports homocysteine elevation, whereas LD administration with inhibitors of the both LD metabolizing enzymes may reduce the risk of homocysteine elevation [39, 46]. Experimental animal studies with a one single LD dose acute treatment paradigm also showed an impact on homocysteine generation even after 120 minutes [47–49]. Combination of COMT-inhibitors with LD/DDI also decreases homocysteine generation as shown with tolcapone [46]. In rats, this was also demonstrated with the COMT inhibitor entacapone, but this is under debate [47]. Observational European nonprospective investigations reported lower homocysteine levels in entacapone-treated patients [50–52]. A prospective clinical study on the effect of entacapone application on homocysteine levels was negative in PD patients. This trial might have been under powered because of the folic acid supplementation in the American and Canadian diet, leading to a milder homocysteine increase than expected [53, 54]. Currently these two COMT inhibitors are available, but their modes of action essentially differ. Tolcapone also acts in the brain, whereas entacapone only operates in the periphery [5, 6, 55]. This pharmacological difference between both compounds warrants a more general discussion; whether peripheral or additional central COMT-inhibition is more suitable to modulate homocysteine synthesis in the long term. Experimental investigations in cell culture models showed that COMT activation caused a sustained homocysteine generation in astrocytes and transport to neurons [26, 56]. Central COMT inhibition may even reduce homocysteine levels in glial and neuronal cells and therefore neurotoxic effects of homocysteine. But exclusive central COMT inhibition may also support central dopamine metabolism via monoaminooxidase (MAO), which generates neuronal oxidative stress [57]. Consequently, one may suggest to perform central COMT inhibition only with central MAO-B blocking to reduce free radical synthesis [3]. However this combination may support glial dopamine metabolism via MAO-A and reduce the glial oxidation capacity of N-methylated tetrahydroisoquinolines (TIQ) to their neuronal neurotoxic cations [58]. But central COMT inhibition may also decrease O-methylation of endogenous TIQ to 1-Methyl-TIQ with their neuronal free radical synthesis reducing properties [58]. Therefore this shift from O-methylation to N-methylation as a consequence of central COMT inhibition may further enhance synthesis N-methylated TIQ, which induces PD [58, 59].

## 7. LD Associated-Homocysteine Increase and Vitamine Supplementation 

Vitmine B complex and folic acid reduce homocysteine, since in example, folic acid catalyses metabolism of homocysteine to methionine [60]. Folic acid intake, which promotes the cobalamin dependent degradation of homocysteine to methionine, or supplementation of B_6_, which supports irreversible metabolism of homocysteine to cysteine, may help [21, 40]. But one animal study showed that folic acid coadministration did not prevent LD-associated hyperhomocysteinemia [48].

## 8. Conclusion and Hypothesis

LD is best available compound for PD treatment. Its delivery to the brain should be more continuous to avoid onset of motor complications. Long term LD application increases homocysteine levels in PD patients. This may contribute to onset of psychiatric side effects, neuronal degeneration, and vascular disease in PD patients. Treatment options for the LD-mediated homocysteine toxicity are vitamin supplementation, that is, folic acid and LD application with COMT inhibition. The possible increased risk for synthesis of free radicals and N-methylated TIQ in association with central COMT inhibition may suggest peripheral COMT inhibition as safer adjunct for LD/DDI therapy.

## References

[1] Fahn S, Oakes D, Shoulson I (2004). Levodopa and the progression of Parkinson's disease. *The New England Journal of Medicine*.

[2] Kann PH (2003). Growth hormone in anti-aging medicine: a critical review. *Aging Male*.

[3] Müller T, Kuhn W, Przuntek H (1993). Therapy with central active catechol-O-methyltransferase (COMT)-inhibitors: is addition of monoamine oxidase (MAO)-inhibitors necessary to slow progress of neurodegenerative disorders?. *Journal of Neural Transmission*.

[4] Zhu BT (2002). Catechol-O-methyltransferase (COMT)-mediated methylation metabolism of endogenous bioactive catechols and modulation by endobiotics and kenobiotics: importance in pathophysiology and pathogenesis. *Current Drug Metabolism*.

[5] Ceravolo R, Piccini P, Bailey DL, Jorga KM, Bryson H, Brooks DJ (2002). 18F-dopa PET evidence that tolcapone acts as a central COMT inhibitor in Parkinson's disease. *Synapse*.

[6] Russ H, Müller T, Woitalla D, Rahbar A, Hahn J, Kuhn W (1999). Detection of tolcapone in the cerebrospinal fluid of parkinsonian subjects. *Naunyn-Schmiedeberg's Archives of Pharmacology*.

[7] Müller T, Erdmann C, Muhlack S, Bremen D, Przuntek H, Woitalla D (2006). Inhibition of catechol-O-methyltransferase contributes to more stable levodopa plasma levels. *Movement Disorders*.

[8] Müller T, Kuhn W (2006). Tolcapone decreases plasma levels of S-adenosyl-L-homocysteine and homocysteine in treated Parkinson's disease patients. *European Journal of Clinical Pharmacology*.

[9] Ben-Shlomo Y, Marmot MG (1995). Survival and cause of death in a cohort of patients with parkinsonism: possible clues to aetiology?. *Journal of Neurology Neurosurgery and Psychiatry*.

[10] Grorell JM, Johnson CC, Rybicki BA (1994). Parkinson's disease and its comorbid disorders: an analysis of Michigan mortality data, 1970 to 1990. *Neurology*.

[11] Miller JW, Shukitt-Hale B, Villalobos-Molina R, Nadeau MR, Selhub J, Joseph JA (1997). Effect of L-DOPA and the catechol-O-methyltransferase inhibitor Ro 41- 0960 on sulfur amino acid metabolites in rats. *Clinical Neuropharmacology*.

[12] Yasui K, Kowa H, Nakaso K, Takeshima T, Nakashima K (2000). Plasma homocysteine and MTHFR C677T genotype in levodopa-treated patients with PD. *Neurology*.

[13] Yasui K, Kowa H, Nakaso K, Takeshima T, Nakashima K (2001). Plasma homocysteine and MTHFR C677T genotype in levodopa-treated patients with PD. *Neurology*.

[14] Müller T, Werne B, Fowler B, Kuhn W (1999). Nigral endothelial dysfunction, homocysteine, and Parkinson's disease. *The Lancet*.

[15] Müller T, Woitalla D, Kuhn W (2003). Benefit of folic acid supplementation in parkinsonian patients treated with levodopa. *Journal of Neurology Neurosurgery and Psychiatry*.

[16] Müller T, Woitalla D, Fowler B, Kuhn W (2002). 3-OMD and homocysteine plasma levels in parkinsonian patients. *Journal of Neural Transmission*.

[17] Chambers JC, McGregor A, Jean-Marie J, Kooner JS (1998). Acute hyperhomocysteinaemia and endothelial dysfunction. *The Lancet*.

[18] Perry IJ (1999). Homocysteine and risk of stroke. *Journal of Cardiovascular Risk*.

[19] Lee E-SY, Chen H, Soliman KFA, Charlton CG (2005). Effects of homocysteine on the dopaminergic system and behavior in rodents. *NeuroToxicology*.

[20] Chung KKK, Dawson VL, Dawson TM, Packer L, Cadenas E (2005). S-Nitrosylation in Parkinson's disease and related neurodegenerative disorders. *Methods in Enzymology Nitric Oxide, Part E*.

[21] Müller T (2008). Role of homocysteine in the treatment of Parkinson's disease. *Expert Review of Neurotherapeutics*.

[22] Chinta SJ, Kumar MJ, Hsu M (2007). Inducible alterations of glutathione levels in adult dopaminergic midbrain neurons result in nigrostriatal degeneration. *Journal of Neuroscience*.

[23] Allain P, Le Bouil A, Cordillet E, Le Quay L, Bagheri H, Montastruc JL (1995). Sulfate and cysteine levels in the plasma of patients with Parkinson's disease. *NeuroToxicology*.

[24] Kuhn W, Roebroek R, Blom H (1998). Elevated plasma levels of homocysteine in Parkinson's disease. *European Neurology*.

[25] Yasui K, Nakaso K, Kowa H, Takeshima T, Nakashima K (2003). Levodopa-induced hyperhomocysteinaemia in Parkinson's disease. *Acta Neurologica Scandinavica*.

[26] Huang G, Dragan M, Freeman D, Wilson JX (2005). Activation of catechol-O-methyltransferase in astrocytes stimulates homocysteine synthesis and export to neurons. *Glia*.

[27] Müller T, Kuhn W (2009). Cysteine elevation in Levodopa-treated patients with Parkinson's disease. *Movement Disorders*.

[28] Hernanz A, de la Fuente M, Navarro M, Frank A (2007). Plasma aminothiol compounds, but not serum tumor necrosis factor receptor II and soluble receptor for advanced glycation end products, are related to the cognitive impairment in Alzheimer's disease and mild cognitive impairment patients. *NeuroImmunoModulation*.

[29] Rogers JD, Sanchez-Saffon A, Frol AB, Diaz-Arrastia R (2003). Elevated plasma homocysteine levels in patients treated with levodopa: association with vascular disease. *Archives of Neurology*.

[30] Sachdev PS, Valenzuela M, Wang XL, Looi JCL, Brodaty H (2002). Relationship between plasma homocysteine levels and brain atrophy in healthy elderly individuals. *Neurology*.

[31] Müller T, Renger K, Kuhn W (2004). Levodopa-associated increase of homocysteine levels and sural axonal neurodegeneration. *Archives of Neurology*.

[32] Duan W, Ladenheim B, Cutler RG, Kruman II, Cadet JL, Mattson MP (2002). Dietary folate deficiency and elevated homocysteine levels endanger dopaminergic neurons in models of Parkinson's disease. *Journal of Neurochemistry*.

[33] Nakaso K, Yasui K, Kowa H (2003). Hypertrophy of IMC of carotid artery in Parkinson's disease is associated with L-DOPA, homocysteine, and MTHFR genotype. *Journal of the Neurological Sciences*.

[34] O'Suilleabhain PE, Sung V, Hernandez C (2004). Elevated plasma homocysteine level in patients with Parkinson disease: motor, affective, and cognitive associations. *Archives of Neurology*.

[35] Ozer F, Meral H, Hanoglu L (2006). Plasma homocysteine levels in patients treated with levodopa: motor and cognitive associations. *Neurological Research*.

[36] Zoccolella S, Lamberti P, Iliceto G (2005). Plasma homocysteine levels in L-dopa-treated Parkinson's disease patients with cognitive dysfunctions. *Clinical Chemistry and Laboratory Medicine*.

[37] Hassin-Baer S, Cohen O, Vakil E (2006). Plasma homocysteine levels and parkinson disease: disease progression, carotid intima-media thickness and neuropsychiatric complications. *Clinical Neuropharmacology*.

[38] O'Suilleabhain PE, Oberle R, Bartis C, Dewey RB, Bottiglieri T, Diaz-Arrastia R (2006). Clinical course in Parkinson's disease with elevated homocysteine. *Parkinsonism and Related Disorders*.

[39] Miller JW, Selhub J, Nadeau MR, Thomas CA, Feldman RG, Wolf PA (2003). Effect of L-dopa on plasma homocysteine in PD patients: relationship to B-vitamin status. *Neurology*.

[40] Woitalla D, Kuhn W, Müller T (2004). MTHFR C677T polymorphism, folic acid and hyperhomocysteinemia in levodopa treated patients with Parkinson's disease. *Journal of Neural Transmission, Supplement*.

[41] Müller T, Erdmann C, Bremen D (2006). Impact of gastric emptying on levodopa pharmacokinetics in Parkinson disease patients. *Clinical Neuropharmacology*.

[42] Muhlack S, Woitalla D, Welnic J, Twiehaus S, Przuntek H, Müller T (2004). Chronic levodopa intake increases levodopa plasma bioavailability in patients with Parkinson's disease. *Neuroscience Letters*.

[43] Woitalla D, Goetze O, Kim JI (2006). Levodopa availability improves with progression of Parkinson's disease. *Journal of Neurology*.

[44] Müller T, Woitalla D, Saft C, Kuhn W (2000). Levodopa in plasma correlates with body weight of parkinsonian patients. *Parkinsonism and Related Disorders*.

[45] Müller T, Kuhn W (2009). Homocysteine levels after acute levodopa intake in patients with Parkinson's disease. *Movement Disorders*.

[46] Müller T, Kuhn W (2006). Tolcapone decreases plasma levels of S-adenosyl-L-homocysteine and homocysteine in treated Parkinson's disease patients. *European Journal of Clinical Pharmacology*.

[47] Nissinen E, Nissinen H, Larjonmaa H (2005). The COMT inhibitor, entacapone, reduces levodopa-induced elevations in plasma homocysteine in healthy adult rats. *Journal of Neural Transmission*.

[48] Henninger N, Wang Q, Okun JG, Schwab S, Krause M (2007). Nitrous oxide promotes hyperhomocysteinemia in levodopa treated rats. *Parkinsonism and Related Disorders*.

[49] Miller JW, Shukitt-Hale B, Villalobos-Molina R, Nadeau MR, Selhub J, Joseph JA (1997). Effect of L-DOPA and the catechol-O-methyltransferase inhibitor Ro 41- 0960 on sulfur amino acid metabolites in rats. *Clinical Neuropharmacology*.

[50] Lamberti P, Zoccolella S, Iliceto G (2005). Effects of levodopa and COMT inhibitors on plasma homocysteine in Parkinson's disease patients. *Movement Disorders*.

[51] Valkovič P, Benetin J, Blažíček P, Valkovičová L, Gmitterová K, Kukumberg P (2005). Reduced plasma homocysteine levels in levodopa/entacapone treated Parkinson patients. *Parkinsonism and Related Disorders*.

[52] Zoccolella S, Lamberti P, Armenise E (2005). Plasma homocysteine levels in Parkinson's disease: role of antiparkinsonian medications. *Parkinsonism and Related Disorders*.

[53] Postuma RB, Espay AJ, Zadikoff C (2006). Vitamins and entacapone in levodopa-induced hyperhomocysteinemia: a randomized controlled study. *Neurology*.

[54] Zesiewicz TA, Wecker L, Sullivan KL, Merlin LR, Hauser RA (2006). The controversy concerning plasma homocysteine in Parkinson disease patients treated with levodopa alone or with entacapone: effects of vitamin status. *Clinical Neuropharmacology*.

[55] Apud JA, Mattay V, Chen J (2007). Tolcapone improves cognition and cortical information processing in normal human subjects. *Neuropsychopharmacology*.

[56] Maler JM, Seifert W, Hüther G (2003). Homocysteine induces cell death of rat astrocytes in vitro. *Neuroscience Letters*.

[57] Gerlach M, Xiao A-Y, Kuhn W (2001). The central catechol-O-methyltransferase inhibitor tolcapone increases striatal hydroxyl radical production in L-DOPA/carbidopa treated rats. *Journal of Neural Transmission*.

[58] Abe K, Saitoh T, Horiguchi Y, Utsunomiya I, Taguchi K (2005). Synthesis and neurotoxicity of tetrahydroisoquinoline derivatives for studying Parkinson's disease. *Biological and Pharmaceutical Bulletin*.

[59] Maruyama W, Abe T, Tohgi H, Dostert P, Naoi M (1996). A dopaminergic neurotoxin, (R)-N-methylsalsolinol, increases in parkinsonian cerebrospinal fluid. *Annals of Neurology*.

[60] Malinow MR, Nieto FJ, Kruger WD (1997). The effects of folic acid supplementation on plasma total homocysteine are modulated by multivitamin use and methylenetetrahydrofolate reductase genotypes. *Arteriosclerosis, Thrombosis, and Vascular Biology*.

